# Prospective Efficacy of Cutotype‐Specific Personalized Topical Postbiotic Emollients in Females: A Single‐Center, Cluster‐Based, Pre‐Post, Nonrandomized Controlled Trial in South Korea

**DOI:** 10.1111/jocd.70171

**Published:** 2025-04-07

**Authors:** Jiyeon Oh, Hyeon Jin Kim, Seoyeon Kyung, Jiseung Kang, So Min Kang, Dasom Jeon, Hye‐Been Kim, HyungWoo Jo, Dong‐Geol Lee, Dong Keon Yon

**Affiliations:** ^1^ Department of Medicine Kyung Hee University College of Medicine Seoul South Korea; ^2^ Center for Digital Health Medical Science Research Institute, Kyung Hee University College of Medicine Seoul South Korea; ^3^ Department of Regulatory Science Kyung Hee University Seoul South Korea; ^4^ Research and Innovation Center Cosmax BTI Seongnam South Korea; ^5^ Department of Anesthesia, Critical Care and Pain Medicine Massachusetts General Hospital Boston Massachusetts USA; ^6^ Division of Sleep Medicine Harvard Medical School Boston Massachusetts USA; ^7^ School of Health and Environmental Science Korea University College of Health Science Seoul South Korea; ^8^ Korea Biomedical Research Institute Seongnam South Korea; ^9^ Department of Microbiology Dankook University Cheonan South Korea; ^10^ Department of Pediatrics Kyung Hee University College of Medicine Seoul South Korea

**Keywords:** cutaneous, cutaneous bioavailability, sensitive skin

To the Editor,

## 1 Introduction

Skin, the human body's largest organ, hosts diverse microbiomes that regulate host immunity and maintain skin physiology [1]. With advancements in metagenomics sequencing [2], our understanding of microbial diversity and composition has significantly expanded. Its composition substantially varies between individuals; therefore, several studies have identified distinct cutotypes, or microbial sub‐community clusters, in various ethnic populations [3]. Our previous study established a comprehensive set of criteria to classify South Koreans' cutotype into 12 groups, considering both biophysical characteristics and microbiome composition [4]. Each of the four skin groups—HL (high in tone/elasticity but low in oil/water), LH (low in tone/elasticity but high in oil/water), LL (low in both), HH (high in both)—is further stratified by age groups (young, 10–34 years; aging, 35–50 years; old, ≥ 51 years) [4].

Given the complex interplay between the application of skincare products and the skin microbiome, it has been a novel objective for skincare products to maintain the skin microbiome. A previous study underscored the positive role of *Staphylococcus* in increasing skin moisture, *Rothia* in mitigating inflammation, *Cutibacterium* in boosting nutrition, and *Streptococcus* in improving elasticity [5]. Skincare products tailored to an individual's cutotype may help address specific skin concerns more effectively by balancing the unique microbial system on the skin. Moreover, supporting the natural balance of the skin microbiome may further contribute to long‐term skin health, maintaining the skin's protective barrier. Thus, we aimed to develop a microbiome postbiotic emollient, with different microbiome compositions for each cutotype, for Korean females with low tone/elasticity (i.e., LL and LH) and evaluate the effects of these products on skin hydration, elasticity, and tightness.

## 2 Methods

### 2.1 Study Setting and Design

This is a prospective, single‐center, and pre‐post cluster nonrandomized controlled trial conducted in South Korea between Jun 2023 and October 2023, following the principles of the Declaration of Helsinki. Written informed consent was collected from all participants before enrollment, and this study was approved by the Ethics Committee of Korea Biomedical Research Institute Institutional Review Board (E‐2023‐010‐01). Participants were enrolled by the Korea Biomedical Research Institute (single center). The study was registered with the Clinical Research Information Service of South Korea (KCT0009958).

A total of 126 healthy females aged 10 years or older were recruited. Inclusion criteria included low skin tone or elasticity (LL and LH cutotypes) without any chronic skin conditions, such as atopic dermatitis or psoriasis. Participants were stratified by cutotype and age group (young, 10–34 years; aging, 35–50 years; old, ≥ 51 years): 22 young LH, 21 aging LH, 21 old LH, 20 young LL, 21 aging LL, and 21 old LL.

### 2.2 Outcomes

We used a questionnaire about skin‐related symptoms to describe the baseline characteristics of participants. We applied the microbiome‐based fermented emollient to all participants but with different compositions based on cutotypes. The microbiome composition was determined to fill the skin elasticity and moisture for each cutotype as follows: *Staphylococcus* (15%) and *Rothia* (15%) for LH and *Cutibacterium* (11%), *Staphylococcus* (6%), and *Streptococcus* (13%) for LL. There was no difference in microbiome composition across age groups within the same type. All microbiomes were sourced from the COSMAX Microbiome Bank (CMB; COSMAX BTI Inc., Seongnam, South Korea) [6]. Participants applied daily cutotype‐specific, topical postbiotic emollients for 2 weeks.

Before and after application of the emollient, we assessed skin hydration (range, 0–120 A.U.; Corneometer CM 825 [Germany]), skin elasticity (range, 0%–100%; Cutometer MPA580 [Germany]), and skin tightness (range, 0°–90°; F‐RAY [South Korea]) for all individuals. We used Image‐Pro 10 (USA) to assess the skin tightness parameter. Higher values indicated improved skin hydration and elasticity, while lower values indicated improved skin tightness.

### 2.3 Sample Size Calculation

Based on our previous large‐scale randomized controlled trial [6], the study originally estimated that the pre‐post group would have 70% power to demonstrate an improvement in skin hydration among participants (4.56 in the placebo group versus 9.37 in the intervention group) at a 5% significance level with a 1:1 ratio. Initially, 19 participants were enrolled in each group. Taking into account a 10% dropout rate, the final sample consisted of 21 participants, considering the nature of the pre‐post setting. Although there were no previous studies on skin tightness and elasticity, based on the expected sample size for skin hydration, it was assumed that the same mechanism would apply, and therefore, the same sample size was designated.

### 2.4 Statistical Analysis

A paired t‐test was performed to assess the effectiveness of microbiome‐based skincare products for each cutotype group, with estimates of mean difference and 95% confidence intervals (95% CIs). We used R statistical software, version 4.4.0 (R Foundation for Statistical Computing), to perform all statistical analyses. A two‐sided *p*‐value < 0.05 was considered statistically significant.

## 3 Results

The mean age of the 126 participants was 45.06 (standardized deviation, 15.19) years, with ages ranging from 20 to 67 years. Baseline characteristics, such as sleep time, sunlight exposure, smoking, and phase of menstrual cycle, are detailed in Table 1. Improvements in skin hydration, elasticity, and tightness were observed across all age groups for both cutotypes (Table 2). The most significant increase in skin hydration was observed in aging LH, with a mean difference of 1.76 (95% CI, 1.25–2.28), whereas the greatest enhancements in elasticity and tightness were found in young LH (elasticity, 0.60 [95% CI, 0.33–0.87]; tightness, −1.03 [95% CI, −1.36 to −0.70]). Similarly, for LL, three age groups showed enhanced skin hydration, elasticity, and tightness. The most considerable increase in skin hydration was found in young LL (4.39 [95% CI, 2.52–6.26]), while aging LL exhibited the most significant enhancements in elasticity and tightness (elasticity, 0.72 [95% CI, 0.22–1.22]; tightness, −0.76 [95% CI, −1.25 to −0.27]). No significant safety problems were reported during the study, although 3.17% (4/126) of participants reported itchy skin after the microbiome emollient.

**TABLE 1:** Baseline characteristics of 126 females.

Continuous variable, mean (SD)	
Age, years	45.06 (15.19)
Continuous variables, *n* (%)	
Cutotype	
Young LH	22 (17.46)
Aging LH	21 (16.67)
Old LH	21 (16.67)
Young LL	20 (15.87)
Aging LL	21 (16.67)
Old LL	21 (16.67)
Sleep time (per day)	
< 4 h	1 (0.79)
4–8 h	111 (88.10)
≥ 8 h	14 (11.11)
Sunlight exposure (per day)	
< 1 h	46 (36.51)
1–4 h	78 (61.90)
≥ 4 h	2 (1.59)
Smoking (pack of cigarettes per day)	
None	121 (96.03)
Half	4 (3.17)
Half to one	1 (0.79)
Itchy skin after the application of the microbiome regimen	
Yes	4 (3.17)
No	122 (96.83)
Skin changes during menstruation	
Yes	27 (21.43)
No	44 (34.92)
Menopause	55 (43.65)
Phase of the menstrual cycle	
Within a week before menstruation	18 (14.29)
During menstruation	8 (6.35)
After a week of menstruation	19 (15.08)
Menopause	56 (44.44)
Unknown	25 (19.84)

Abbreviations: LH, low in tone/elasticity and high in oil/water; LL, low in tone/elasticity and oil/water; SD, standard deviation.

**TABLE 2:** Mean difference of skin hydration, elasticity, and tightness between before‐ and after‐test.

Variables	Pre‐test, mean (95% CI)	Post‐test, mean (95% CI)	Difference between pre‐test and post‐test	
			Mean difference (95% CI)	*p*
Young LH (*n* = 22)				
Skin hydration	58.20 (52.62 to 63.78)	59.13 (53.55 to 64.71)	**0.93 (0.51 to 1.36)**	**< 0.001**
Skin elasticity	75.10 (73.20 to 77.01)	75.70 (73.80 to 77.60)	**0.60 (0.33 to 0.87)**	**< 0.001**
Skin tightness	40.59 (38.49 to 42.68)	39.56 (37.57 to 41.54)	**−1.03 (−1.36 to − 0.70)**	**< 0.001**
Aging LH (*n* = 21)				
Skin hydration	60.97 (56.76 to 65.19)	62.74 (58.38 to 67.10)	**1.76 (1.25 to 2.28)**	**< 0.001**
Skin elasticity	73.71 (71.79 to 75.64)	74.07 (72.13 to 76.00)	**0.35 (0.25 to 0.46)**	**< 0.001**
Skin tightness	37.42 (34.95 to 39.90)	36.63 (34.07 to 39.18)	**−0.80 (−1.25 to − 0.35)**	**0.002**
Old LH (*n* = 21)				
Skin hydration	54.29 (51.82 to 56.76)	55.12 (52.71 to 57.53)	**0.83 (0.57 to 1.08)**	**< 0.001**
Skin elasticity	71.53 (69.71 to 73.34)	71.75 (69.90 to 73.60)	**0.22 (0.14 to 0.31)**	**< 0.001**
Skin tightness	33.29 (31.26 to 35.32)	32.90 (30.88 to 34.91)	**−0.40 (−0.46 to − 0.33)**	**< 0.001**
Young LL (*n* = 20)				
Skin hydration	55.12 (52.07 to 58.16)	59.51 (55.92 to 63.10)	**4.39 (2.52 to 6.26)**	**< 0.001**
Skin elasticity	75.83 (74.10 to 77.56)	76.38 (74.62 to 78.13)	**0.54 (0.26 to 0.83)**	**< 0.001**
Skin tightness	38.43 (36.80 to 40.06)	37.80 (36.13 to 39.47)	**−0.63 (−1.10 to − 0.17)**	**0.011**
Aging LL (*n* = 21)				
Skin hydration	49.79 (47.29 to 52.29)	50.71 (48.30 to 53.12)	**0.92 (0.50 to 1.35)**	**< 0.001**
Skin elasticity	69.50 (67.30 to 71.71)	70.23 (68.16 to 72.30)	**0.72 (0.22 to 1.22)**	**0.007**
Skin tightness	34.25 (31.65 to 36.85)	33.49 (30.85 to 36.13)	**−0.76 (−1.25 to − 0.27)**	**0.004**
Old LL (*n* = 21)				
Skin hydration	47.76 (45.39 to 50.14)	48.48 (45.97 to 50.99)	**0.71 (0.36 to 1.07)**	**< 0.001**
Skin elasticity	70.12 (68.35 to 71.89)	70.45 (68.70 to 72.20)	**0.33 (0.17 to 0.49)**	**< 0.001**
Skin tightness	39.40 (36.10 to 42.70)	39.14 (35.84 to 42.43)	**−0.26 (−0.33 to − 0.19)**	**< 0.001**

*Note:* Number in bold indicates a significant difference (*p* < 0.05).

Abbreviations: CI, confidence interval; LH, low in tone/elasticity and high in oil/water; LL, low in tone/elasticity and oil/water.

## 4 Discussion

This study showed the efficacy of microbiome‐based emollients tailored to specific cutotypes in improving skin hydration, elasticity, and tightness. The results underscore the potential therapeutic applications of such products in personalized skincare. However, these findings should be interpreted with caution due to several limitations. The lack of investigation into changes in the microbiome composition hinders the direct assessment of this emollient's impact on the skin microbiome. In addition, we only recruited females, limiting our ability to generalize the findings to broader applications. Moreover, due to the nature of the pre–post‐test study design, selection bias, and time‐related bias may influence the changes observed between pre‐ and post‐tests. Last, although the short duration of the intervention (2 weeks) was chosen to observe initial short‐term effects, longer‐term studies are needed to assess sustained outcomes.

## 5 Conclusion

Based on our novel classification of skin types, we developed skin care products for each cutotype. The microbiome skin care products customized for LL and LH cutotypes appear safe and effective, with significant improvements in skin hydration, elasticity, and tightness across all cutotype groups; however, the short intervention period and small sample size limit conclusions on long‐term effects, warranting further research. This study may serve as a milestone for future studies investigating the association between cutotype and skin health.

## Author Contributions

D.K.Y. had full access to all of the data in the study and took responsibility for the integrity of the data and the accuracy of the data analysis. All authors approved the final version of the manuscript before submission. Study concept and design: J.O., H.J.K., D‐.G.L., and D.K.Y.; acquisition, analysis, or interpretation of data: J.O., H.J.K., D‐.G.L., and D.K.Y.; drafting of the manuscript: J.O., H.J.K., D‐.G.L., and D.K.Y.; critical revision of the manuscript for important intellectual content: all authors; statistical analysis: J.O., H.J.K., D‐.G.L., and D.K.Y.; study supervision: D.K.Y. D.K.Y. supervised the study and served as a guarantor. J.O. and H.J.K. contributed equally. H.J., D‐.G.L., and D.K.Y. contributed equally as corresponding authors. The corresponding author attests that all listed authors meet the authorship criteria and that no one meeting the criteria has been omitted.

## Conflicts of Interest

The authors declare no conflicts of interest.

## Data Availability

The data supporting this study's findings are available from the corresponding author upon reasonable request.
